# Cytokine signatures of *Plasmodium vivax* infection during pregnancy and delivery outcomes

**DOI:** 10.1371/journal.pntd.0008155

**Published:** 2020-05-04

**Authors:** Carlota Dobaño, Azucena Bardají, Myriam Arévalo-Herrera, Flor E. Martínez-Espinosa, Camila Bôtto-Menezes, Norma Padilla, Michela Menegon, Swati Kochar, Sanjay Kumar Kochar, Holger Unger, Maria Ome-Kaius, Anna Rosanas-Urgell, Adriana Malheiros, Maria Eugenia Castellanos, Dhiraj Hans, Meghna Desai, Aina Casellas, Chetan E. Chitnis, Carlo Severini, Ivo Mueller, Stephen Rogerson, Clara Menéndez, Pilar Requena

**Affiliations:** 1 ISGlobal, Hospital Clínic—Universitat de Barcelona, Barcelona, Catalonia, Spain; 2 Caucaseco Scientific Research Center, Cali, Colombia; 3 Fundação de Medicina Tropical Dr. Heitor Vieira Dourado, Manaus—AM, Brazil; 4 Instituto Leônidas e Maria Deane, Adrianópolis, Manaus, Brazil; 5 Universidade do Estado do Amazonas, Cachoeirinha, Manaus—AM, Brazil; 6 Centro de Estudios en Salud, Universidad del Valle de Guatemala, Guatemala, Guatemala; 7 Istituto Superiore di Sanità, Rome, Italy; 8 SP Medical College, PBM Hospital, Bikaner, India; 9 Papua New Guinea Institute of Medical Research, Madang, Papua New Guinea; 10 Department of Biomedical Sciences, Institute of Tropical Medicine, Antwerp, Belgium; 11 Instituto de Ciências Biológicas, Universidade Federal do Amazonas, Manaus—AM, Brazil; 12 International Center for Genetic Engineering and Biotechnology, New Delhi, India; 13 Malaria Branch, Division of Parasitic Diseases and Malaria, Centers for Disease Control and Prevention, Atlanta, Georgia, United States of America; 14 Malaria Parasite Biology and Vaccines Unit, Institut Pasteur, Paris, France; 15 Walter and Eliza Hall Institute, Parkville, VIC, Australia; 16 University of Melbourne, Parkville, VIC, Australia; 17 Universidad de Granada, Departamento de Medicina Preventiva y Salud Pública, Facultad de Farmacia, Granada, Spain; University of Florida, UNITED STATES

## Abstract

*Plasmodium vivax* malaria is a neglected disease, particularly during pregnancy. Severe vivax malaria is associated with inflammatory responses but in pregnancy immune alterations make it uncertain as to what cytokine signatures predominate, and how the type and quantity of blood immune mediators influence delivery outcomes. We measured the plasma concentrations of a set of thirty-one biomarkers, comprising cytokines, chemokines and growth factors, in 987 plasma samples from a cohort of 572 pregnant women from five malaria-endemic tropical countries and related these concentrations to delivery outcomes (birth weight and hemoglobin levels) and malaria infection. Samples were collected at recruitment (first antenatal visit) and at delivery (periphery, cord and placenta). At recruitment, we found that *P*. *vivax*–infected pregnant women had higher plasma concentrations of proinflammatory (IL-6, IL-1β, CCL4, CCL2, CXCL10) and T_H_1-related cytokines (mainly IL-12) than uninfected women. This biomarker signature was essentially lost at delivery and was not associated with birth weight nor hemoglobin levels. Antiinflammatory cytokines (IL-10) were positively associated with infection and poor delivery outcomes. CCL11 was the only biomarker to show a negative association with *P*. *vivax* infection and its concentration at recruitment was positively associated with hemoglobin levels at delivery. Birth weight was negatively associated with peripheral IL-4 levels at delivery. Our multi-biomarker multicenter study is the first comprehensive one to characterize the immunological signature of *P*. *vivax* infection in pregnancy thus far. In conclusion, data show that while T_H_1 and pro-inflammatory responses are dominant during *P*. *vivax* infection in pregnancy, antiinflammatory cytokines may compensate excessive inflammation avoiding poor delivery outcomes, and skewness toward a T_H_2 response may trigger worse delivery outcomes. CCL11, a chemokine largely neglected in the field of malaria, emerges as an important marker of exposure or mediator in this condition.

## Author summary

Cytokine and growth factor plasma concentrations were evaluated in women from five countries endemic for malaria *vivax*, at different moments and blood compartments during pregnancy. *P*. *vivax* infection during pregnancy was associated with a pro-inflammatory and T_H_1 response, together with an antiinflammatory response. Nevertheless, at delivery most associations between cytokines and infection were lost. Of note, CCL11/eotaxin, a chemokine not generally analyzed in malaria studies, presented a lower concentration in *P*. *vivax*-infected women and a protective association with hemoglobin levels at delivery. Moreover, IL-4 levels had a negative association with birth weight. Data suggest that a compensated inflammatory/antiinflammatory response in *P*. *vivax* infection during pregnancy might avoid poor delivery outcomes, while a predominance of T_H_2 responses may be detrimental for birth weight. Further research is warranted to unravel the role of CCL11 in malaria infection or exposure.

## Introduction

Malaria caused by *Plasmodium vivax* (*Pv*) is a neglected tropical disease, especially during pregnancy, of worldwide distribution [1]. The negative effects of malaria in pregnant women and their offspring have been better described for malaria caused by *P*. *falciparum* (*Pf*) whereas fewer reports have investigated the outcomes of *Pv* infection in pregnancy [2]. To address this gap, we performed a multicenter cohort study (the PregVax project) to characterize the burden and health consequences of malaria caused by *Pv* in pregnant women from five malaria endemic areas [3]. Within that cohort, we set out to characterize in more depth and breadth the immune responses induced in pregnant women when infected or exposed to *Plasmodium* parasites [4–7], and how they might correlate with negative clinical outcomes.

As part of this investigation, here we aim to better understand the cellular immune mediators circulating in the blood of pregnant women, whose immune system is altered due to pregnancy [8] when facing a parasite infection like *Pv* that has been associated to inflammation, particularly in the case of severe disease [9]. A recent study by Singh et al [10] has shown increased levels of inflammatory markers in vivax malaria during pregnancy, but it only included three cytokines (IL-6, IL-1β and TNF). For a more comprehensive evaluation of the multiple effects that *Pv* infection may elicit in the immune system of pregnant women, a wider set of cellular biomarkers of different functions, including chemokines and growth factors as well as T helper (T_H_)-related and regulatory cytokines, needs to be studied. This is particularly relevant to further understand the role of CCL11 during pregnancy and its association with *Pv* infection, as we previously showed decreased blood concentrations of this chemokine in pregnant women compared to non-pregnant individuals and in malaria-exposed compared to malaria-naïve individuals [7].

In an initial recent analysis, we evaluated the effect of pregnancy and of residing in tropical countries, where exposure to infectious diseases is more common, on the concentration of cytokines in plasma samples from women at different times during gestation and after puerperium, as well as in the three blood compartments (periphery, cord, placenta) [8]. We found that the concentrations of circulating cytokines were the highest at postpartum (at least 10 weeks after delivery), with higher values at delivery compared to the first antenatal clinic visit. Furthermore, anti-plasmodial antibodies (markers of malaria exposure) correlated with cytokine concentrations postpartum, but not during pregnancy, suggesting that pregnancy had a greater effect than malaria exposure on cytokine levels. Additionally, no strong associations between cytokines and gestational age were detected.

In the present study, we assess the relationships between cytokine, chemokine and growth factor plasma concentrations, delivery outcomes and presence of *Pv*, in the PregVax cohort. Our multi-biomarker multicenter study is the first one to characterize the immunological signature of *Pv* infection in pregnancy to this extent.

## Materials and methods

### Study design and population

This analysis was done in the context of the PregVax project, a cohort study of 9,388 pregnant women from five countries where malaria is endemic: Brazil (BR), Colombia (CO), Guatemala (GT), India (IN) and Papua New Guinea (PNG), enrolled between 2008 and 2012 at the first antenatal visit, and followed up until delivery. A venous blood sample was collected to perform immunological assays in the following participants: a) any women with *Pv* infection (with or without *Plasmodium* coinfections) at any visit from any country, b) a random subcohort (approximately 10% of total cohort) assigned as the immunology cohort, at enrolment and delivery. Bleedings at delivery included peripheral, cord and placental (only CO and PNG) blood.

*Pv* and *Pf* (studied as a possible confounder in co-infected women) parasitaemias were assessed at every visit in Giemsa-stained blood slides that were read onsite. An external validation of parasitemia results was performed by expert microscopists in a subsample of slides (100 per country) at the Hospital Clinic and at the Hospital Sant Joan de Deu in Barcelona, Spain. Submicroscopic *Pv* and *Pf* infections were also determined at enrolment and delivery by real time-PCR in a group of participants, which included the immunological subcohort. Malaria symptoms and hemoglobin (Hb, g/dL) levels were also recorded at enrolment and delivery, as well as neonatal birth weight (g).

The protocol was approved by the national and/or local ethics committees of each site, the CDC IRB (USA) and the Hospital Clinic Ethics Review Committee (Barcelona, Spain). Written informed consent was obtained from all study participants. All human subjects were adults.

### Isolation of plasma

Five to 10 mL of blood were collected aseptically in heparinized tubes. Plasma was separated by centrifuging at 600 g for 10 min at room temperature, aliquoted and stored at -80ºC. Samples from BR, CO, GT and PNG were shipped to the Barcelona Institute for Global Health on dry ice. The measurement of cytokines, chemokines and growth factors (hereinafter together referred to as biomarkers) was performed at ISGlobal, Barcelona (Spain) to minimize inter-site variability. Samples from India were analyzed at ICGEB, Delhi.

### Multiplex bead array assay

The biomarkers were analyzed in thawed plasmas with a multiplex suspension detection system *Cytokine Magnetic 30-Plex Panel* (Invitrogen, Madrid, Spain) which allows the detection of the following biomarkers: epidermal growth factor (EGF), Eotaxin/CCL11, fibroblast growth factor (FGF), granulocyte colony-stimulating factor (G-CSF), granulocyte-macrophage colony-stimulating factor (GM-CSF), hepatocyte growth factor (HGF), interferon (IFN)-α, IFN-γ, interleukin (IL)-1RA, IL-1β, IL-2, IL-2R, IL-4, IL-5, IL-6, IL-7, IL-8/CXCL8, IL-10, IL-12(p40/p70), IL-13, IL-15, IL-17, IFN-γ induced protein (IP-10/CXCL10), monocyte chemoattractant protein (MCP-1/CCL2), monokine induced by IFN-γ (MIG/CXCL9), macrophage inflammatory protein (MIP)-1α/CCL3, MIP-1β/CCL4, regulated on activation, normal T cell expressed and secreted (RANTES/CCL5), tumor necrosis factor (TNF), and vascular endothelial growth factor (VEGF). Fifty μL of the plasmas were tested in single replicates (dilution 1:2, as recommended by the vendor). Each plate contained serial dilutions (1:3) of a standard sample of known concentration of each analyte provided by the manufacturer, as well as a blank control and a reference sample control for quality control purposes, all of them in duplicates. Upper and lower values of the standard curves for each analyte are displayed in S1 Table.

The assays were carried out according to the manufacturer’s instructions. Beads were acquired on the BioPlex100 system (Bio-Rad, Hercules, CA) and concentrations calculated using the Bioplex software. When values were out of range (OOR) according to the software, a value three-times lower than the lowest standard concentration was assigned (as standard dilutions were 1:3) for OOR values under the curves, and a value three-times higher than the highest standard concentration was assigned for OOR values above the curve. Moreover, the software extrapolated values below and above the lower and higher concentrations, respectively, of the standard curves when they fitted into the curves and were not OOR. These values were kept with the exception of those three-times below the lowest standard concentration and three-times above the highest standard concentration, for which those respective values were assigned.

In addition, the cytokine TGF-β1 was analyzed in all plasmas except those from India, with a DuoSet ELISA kit (R&D Systems). Following the vendor’s recommendations, latent TGF-β1 was activated to its immunoreactive form with HCl and neutralized with NaOH/HEPES. A 40-fold plasma dilution was used.

### *Plasmodium* spp. detection by real time-PCR

From the whole cohort (9,388 women), 1500 recruitment and 1500 different delivery samples were randomly selected for PCR. Samples from BR, CO, GT, and half of the samples from PNG, were analyzed at the *Istituto Superiore di Sanità* (Rome, Italy), as described [3]. The threshold for positivity for each species was established as a cycle threshold <45, according to negative controls. *Pv* diagnosis for IN samples was performed in Delhi following Rome’s protocol adapted for the instrument sensitivity (the third step amplification 72ºC for 25 sec instead of 5 sec). Approximately half of the PNG samples were analyzed for submicroscopic infections in Madang, following a protocol [11] similar to that from Rome, except that the threshold for positivity for each species was established as cycle threshold <40, according to negative controls. DNA was extracted from whole blood-spot filter paper.

### Sample selection and statistical methods

It was not possible to measure the biomarkers in all the plasma samples available due to budget constraints. Therefore, an initial 50 enrolment samples per country (35 in IN, 235 total) and their paired delivery samples were randomly selected. However, follow-up rates were low and when paired recruitment/delivery samples available were <50, random delivery samples were included to achieve N = 50 (35 in IN). Thus, 129/235 delivery samples were paired to recruitment samples. In addition, because malaria prevalence was generally low in our random subset, we performed a case-control selection including all the available samples from women with a *Pv* infection (diagnosed by microscopy and/or PCR) at recruitment (N = 49) or delivery (N = 18) and similar numbers of randomly-selected samples with a negative *Pv* PCR result, matched by country (N = 62 and N = 7 respectively). Finally, 144 placental plasmas, 125 peripheral plasmas collected at delivery and paired to the placental samples and 112 cord plasmas were analysed. A flow chart with all samples analysed is provided in S1 Fig. Data from the 5 countries were combined in the analysis. Except when otherwise specified, *Pv* infection was defined as either a positive smear or a PCR positive result (or both).

Overall, our aim was to search for the associations between cytokine concentrations and health outcomes (malaria infection, Hb levels and birth weight). In this regard, we performed two separate investigations. First, a cross-sectional analysis in which health outcomes and cytokine concentrations were examined at the same timepoint, either recruitment or delivery. Second, a longitudinal analysis of the effect of cytokine concentrations at recruitment on health outcomes at delivery.

To study the association of biomarkers with *Pv* infection, first a principal component analysis (PCA) was performed. In the PCA, a large set of possibly correlated variables (e.g. cytokines) is transformed into a small set of linearly uncorrelated variables called principal components (PC), which may be interpreted as clusters of cytokines. For each PC, we show the contribution (loading score) of each cytokine, considering the generally accepted cut-off established at loading score = 0.3. For further analyses, we only considered the seven PCs that accounted more for the variance of the data set (Eigenvalue ≥1, Kaiser-Guttman criterion). To use PCs as variables (each representing several cytokines at a time), PC scores were predicted for each subject and logistic regression models were estimated with PCs as independent variables and *Pv* infection as the dependent variable. We excluded TGF-β from the PCA analysis as this cytokine was analyzed by a different technique (ELISA) and was not measured in IN. Finally, to assess if our data set was suitable for PCA, we ran the Kaiser-Meyer-Olkin (kmo) test for sampling adequacy.

To assess the association between individual biomarker concentrations and *Pv* infection, we used the Mann-Whitney test in the crude analysis (corrected for multiple comparisons with the Benjamini-Hochberg method) and estimated multivariable logistic regression models adjusting for the following variables: site, age at recruitment, gestational age, parity, delivery mode (vaginal vs caesarean birth) for the analysis with cytokine concentration at delivery and *Pf* infection. At delivery, three different blood compartments were investigated: periphery, placenta and cord. For this objective, pairwise statistical significance was interpreted based on 95% confidence intervals (CI), and considered significant when the interval did not include 1. The same adjusted regression model was estimated to analyze the association between peripheral plasma biomarker concentration at recruitment and future (at delivery) *Pv* infection. Furthermore, the association of biomarkers with submicroscopic and microscopic *Pv* infections was assessed with linear regression models adjusted by site. Finally, the association between biomarker concentrations in plasma with maternal Hb levels and birth weight was assessed using multivariable linear regression models, adjusted for site, age (at recruitment), Hb levels (at recruitment), parity, delivery mode for the analysis with cytokine concentration at delivery, *Pv* and *Pf* infection during pregnancy. For this objective, pairwise statistical significance was interpreted based on 95% confidence intervals, and considered significant when the interval did not include 0.

Overall, significance was defined at p<0.05. Analyses and graphs were performed using Stata/SE 10.1 (College Station, TX, USA) and GraphPad Prism (La Jolla, CA, USA).

## Results

### Characteristics of the study population

A total of 987 plasma samples belonging to 572 pregnant women were analyzed for biomarker concentration, comprising 346 peripheral plasma samples collected at recruitment, 385 peripheral plasmas at delivery, 112 cord plasmas and 144 placental plasmas. Unfortunately, only 129 samples were paired between recruitment and delivery due to low follow-up rates. The study population characteristics at baseline are provided in Table 1. The number of infection cases by country, method and timepoint are provided in S2 Table.

**Table 1:** Baseline characteristics of study population. This refers to all the women included in the study at both timepoints.

		Site				
		Brazil N = 90	Colombia N = 116	Guatemala N = 91	India N = 58	PNG N = 217
**Age (years)**^a^^,^^b^		23.3 (6.0) [90]	22.3 (5.8) [115]	25.0 (7.7) [91]	23.5 (3.5) [58]	25.5 (5.7) [210]
**Gravidity (number of previous pregnancies)**^d^	0	26 (29%)	34 (30%)	30 (33%)	27 (47%)	69 (38%)
	1–3	42 (47%)	56 (49%)	31 (34%)	29 (50%)	76 (42%)
	4+	22 (24%)	25 (21%)	30 (33%)	2 (3%)	37 (20%)
**GA at recruitment** **(weeks)**^d^	0–12	15 (17%)	28 (24%)	5 (6%)	1 (2%)	11 (6%)
	13–24	30 (34%)	42 (37%)	32 (36%)	30 (52%)	87 (48%)
	25+	43 (49%)	45 (39%)	52 (58%)	27 (47%)	84 (46%)
**GA at delivery (weeks, by Ballard method)**^d^	0–37	5 (8%)	29 (39%)	6 (10%)	29 (69%)	49 (33%)
	38–41	54 (92%)	42 (57%)	35 (58%)	13 (31%)	83 (56%)
	42+	0 (0%)	3 (4%)	19 (32%)	0 (0%)	16 (11%)
**BMI (kg/m**^**2**^**)** ^a^^,^^b^		25.7 (4.4) [89]	23.5 (3.5) [114]	25.8 (3.9) [91]	23.1 (4.5) [58]	23.7 (3.3) [173]
**Hemoglobin (g/dL)**^a^^,^^b^		11.3 (1.3) [90]	10.9 (1.6) [114]	11.1 (1.5) [82]	9.5 (1.6) [58]	9.52 (1.5) [214]
**Birth weight** ^a^^,^^g^ **(g)**		3166.1 (526.4) [61]	3224.11 (408.7) [82]	3151.9.6 (537.3) [60]	3031.7 (436.5) [42]	2923.1 (493.3) [159]
**Delivery mode**^d^	**V**	52 (79)	66 (82)	44 (71)	34 (79)	135 (100)
	**C**	14 (21)	15 (18)	18 (29)	9 (21)	0 (0)
**Syphilis screening**^d^	**POS**	0 (0)	7 (10)	N/A	0 (0)	5 (4)
	**NEG**	56 (100)	64 (90)	N/A	13 (100)	123 (96)

^a^ Arithmetic Mean (standard deviation) [number].

^b^ At recruitment.

^c^ One-way ANOVA.

^d^ n (percentage).

^e^ Chi-squared test.

^f^ Fisher’s exact test.

^g^ birth weight excluding twins. PNG: Papua New Guinea. GA: gestational age (weeks). BMI: body mass index. V: vaginal. C: cesarean section. POS: positive. NEG: negative. N/A: not available.

### Association of *Pv* infection with plasma biomarker concentration at recruitment

Considering the large amount of cytokine variables, we first performed a PCA to reduce the dimensionality of data. The kmo test for sampling adequacy to PCA analysis resulted in kmo = 0.85 that according to the literature might be considered as meritorious [12]. In the PCA analysis, seven PCs contributed mostly to the variance of the data (S3 Table) and were further considered for regression analyses. Of those seven PCs, three had a positive association with *Pv* infection: PC3, PC5 and PC7 (S4 Table). Then we analyzed which cytokines contributed mostly to those PCs associated with *Pv* infection (Table 2). The PC3-proinflammatory-chemokine group showed the highest contribution by CXCL8, CCL4 and CCL3. The PC5-antiinflammatory-inflammatory group had the highest contribution by IL-10, CXCL10, IL-6 and CCL2, whereas the PC7-CCL5-T_H_ group had the highest contribution by CCL5, IL-4, IL-2R and IL-12 (Table 2).

**Table 2:** Loading scores for principal component analysis at recruitment.

Variable	PC1	PC2	PC3	PC4	PC5	PC6	PC7	Unexplained
TNF		0.429	** **		** **		** **	0.271
IL-1β		0.344	** **		** **		** **	0.261
IL-6			** **		**0.356**		** **	0.211
IL-10			** **		**0.506**		** **	0.334
IL-1RA			** **		** **		** **	0.179
IFN-α			** **		** **		** **	0.316
CXCL8			**0.565**		** **		** **	0.271
CCL3			**0.406**		** **		** **	0.262
CCL4			**0.426**		** **		** **	0.267
CCL2			** **		**0.312**		** **	0.290
CXCL10			** **		**0.489**		** **	0.337
CXCL9			** **		** **	0.474	** **	0.439
CCL11			** **		** **	0.634	** **	0.376
CCL5			** **		** **		**0.695**	0.247
IFN-γ			** **	0.487	** **		** **	0.426
IL-12			** **		** **		**0.341**	0.317
IL-2	0.494		** **		** **		** **	0.218
IL-15	0.351		** **		** **		** **	0.319
IL-2R			** **		** **		**0.352**	0.361
IL-4			** **		** **		**0.392**	0.552
IL-5		0.456	** **		** **		** **	0.229
IL-13			** **	0.447	** **		** **	0.377
IL-17		0.468	** **		** **		** **	0.244
EGF	0.373		** **		** **		** **	0.371
FGF	0.497		** **		** **		** **	0.224
HGF			** **		** **		** **	0.403
VEGF			** **		** **		** **	0.495
G-CSF	** **		** **	0.456	** **		** **	0.487
GM-CSF	** **		** **		** **		** **	0.347
IL-7	** **	0.405	** **		** **		** **	0.250

Loading scores for each principal component (PC) with Eigenvalue>1 and proportion of unexplained variance after varimax rotation. In bold the PC with a positive association with *P*. *vivax* infection. Only shown if loading score >0.3.

**Fig 1:**
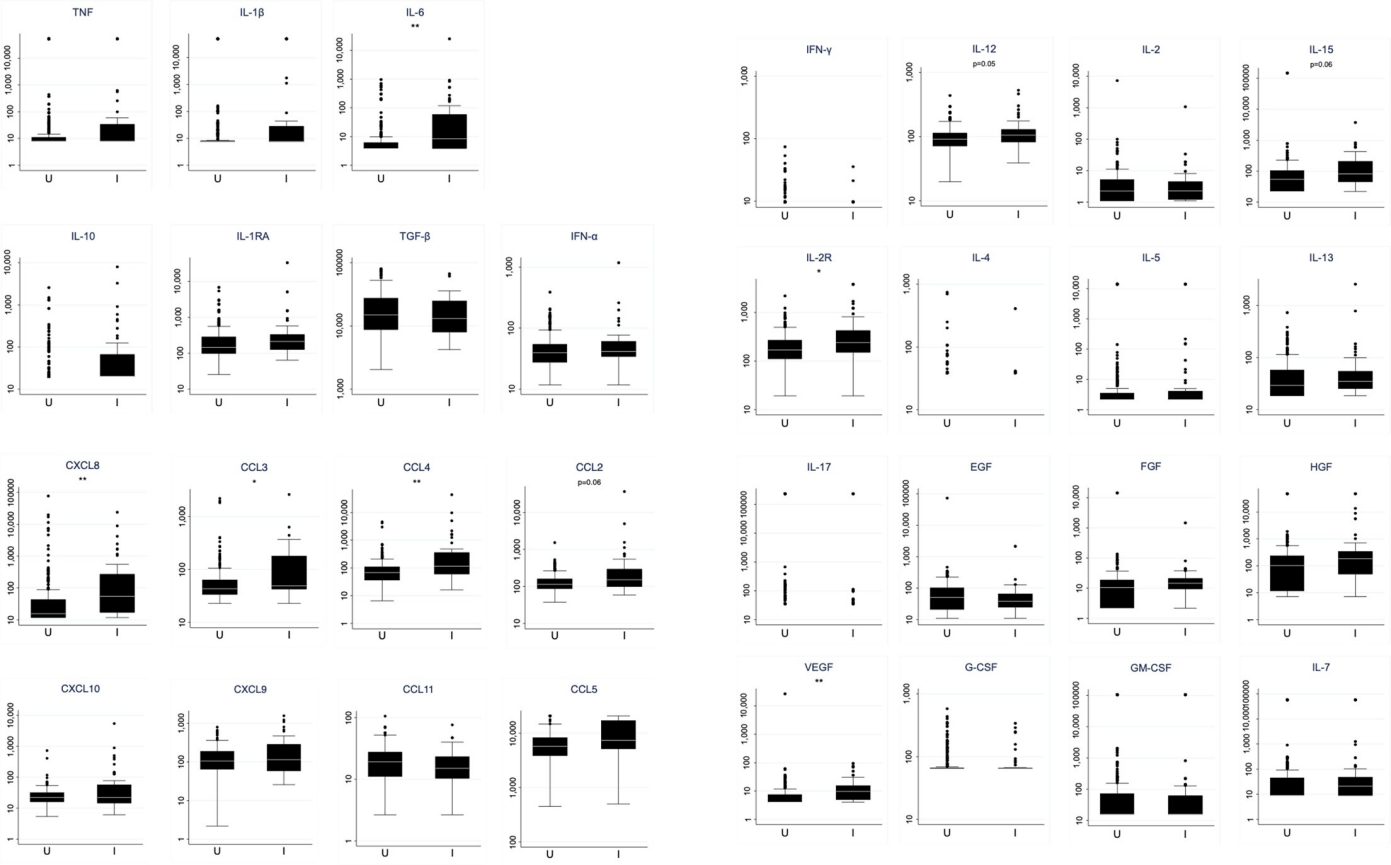
Effect of *Plasmodium vivax* infection on peripheral plasma biomarker concentrations at recruitment. Box plots represent median (white line), and 25^th^ and 75^th^ percentiles (lower and upper hinge respectively) of biomarker concentrations in peripheral plasma at recruitment, in *P*. *vivax* infected (I, N = 54) and uninfected (U, N = 247) pregnant women. Concentrations for all biomarkers are expressed in pg/mL. P-value corresponds to the Mann-Whitney test corrected for multiple comparisons with the Benjamini-Hochberg method. *p<0.05, **p<0.01, ***p<0.001.

As the PCA is quite exploratory, we also analyzed biomarkers individually. We found that *Pv*–infected women had higher plasma concentrations of proinflammatory biomarkers IL-6, CXCL8, CCL3, CCL4 and CCL2, of T_H_1-related cytokines IL-12, IL-15 and IL-2R, and of growth factor VEGF (Fig 1) than uninfected women, consistent with PCA analysis. After adjusting for other confounders (see materials and methods), we found a positive association of *Pv* infection with proinflammatory biomarkers IL-6, IL-1β, CCL4, CCL2, CXCL10 and TNF (borderline non-significant for the latter); the antiinflammatory IL-10; the chemokine CCL5; the T_H_1-related cytokines IL-12 and IL-2R; the T_H_2-related cytokine IL-5; and the growth factors FGF, HGF, VEGF and IL-7 (Table 3). In contrast, a negative association was observed with CCL11 plasma concentration (Table 3).

**Table 3:** Association of plasma biomarker concentration with *P*. *vivax* infection.

	Recruitment		Delivery					
	Periphery		Periphery		Placenta		Cord	
	OR	95%CI	OR	95%CI	OR	95%CI	OR	95%CI
TNF	1.03	1.00; 1.06	1.00	0.88; 1.12	1.02	0.89; 1.18	1.02	0.97; 1.08
IL-1β	**1.06**	**1.02; 1.09**	1.00	0.89; 1.13	1.06	0.93; 1.21	1.00	0.95; 1.05
IL-6	**1.09**	**1.03; 1.15**	0.96	0.87; 1.05	0.91	0.81; 1.04	1.04	0.98; 1.10
IL-10	**1.09**	**1.02; 1.17**	0.97	0.86; 1.09	**1.17**	**1.02; 1.34**	1.06	0.54; 2.07
IL-1RA	1.06	0.98; 1.15	1.06	0.97; 1.16	1.04	0.94; 1.16	1.11	0.94; 1.30
TGF-β	1.01	0.88; 1.17	1.20	0.97; 1.47	1.01	0.87; 1.16	0.91	0.79; 1.06
IFN-α	1.05	0.93; 1.19	**1.33**	**1.11; 1.58**	1.09	0.94; 1.26	1.15	0.78; 1.70
CXCL8	1.02	0.98; 1.07	1.03	0.98; 1.08	0.99	0.92; 1.08	1.05	1.00; 1.11
CCL3	1.04	0.95; 1.13	1.08	0.94; 1.24	0.89	0.74; 1.07	1.06	0.87; 1.28
CCL4	**1.09**	**1.02; 1.17**	0.90	0.77; 1.04	1.01	0.91; 1.12	1.06	0.98; 1.15
CCL2	**1.24**	**1.10; 1.41**	1.01	0.93; 1.09	1.06	0.97; 1.16	1.07	0.99; 1.16
CXCL10	**1.17**	**1.06; 1.29**	1.04	0.91; 1.18	0.93	0.79; 1.09	0.97	0.82; 1.13
CXCL9	1.06	0.97; 1.15	1.03	0.94; 1.14	1.05	0.92; 1.19	1.01	0.92; 1.12
CCL11	**0.88**	**0.77; 0.99**	1.01	0.87; 1.18	0.93	0.80; 1.09	0.91	0.76; 1.08
CCL5	**1.13**	**1.01; 1.27**	1.08	0.93; 1.26	0.93	0.84; 1.02	1.01	0.84; 1.22
IFN-γ	1.16	0.84; 1.59	1.38	0.89; 2.14	1.00	N/A	0.56	0.01; 36.96
IL-12	**1.37**	**1.11; 1.68**	**1.57**	**1.15; 2.15**	0.97	0.77; 1.23	0.84	0.60; 1.18
IL-2	1.04	0.97; 1.11	0.98	0.89; 1.09	1.06	0.96; 1.17	1.03	0.95; 1.11
IL-15	1.03	0.98; 1.08	1.03	0.91; 1.16	1.05	0.93; 1.20	0.97	0.83; 1.13
IL-2R	**1.12**	**1.02; 1.24**	0.99	0.87; 1.14	0.91	0.75; 1.09	0.94	0.76; 1.15
IL-4	0.94	0.73; 1.22	1.04	0.83; 1.31	0.92	0.52; 1.64	0.69	0.06; 8.61
IL-5	**1.04**	**1.01; 1.08**	1.00	0.91; 1.11	1.15	0.87; 1.51	1.00	0.95; 1.06
IL-13	1.10	0.99; 1.22	0.98	0.86; 1.12	0.91	0.70; 1.19	0.84	0.57; 1.22
IL-17	1.03	0.99; 1.07	1.10	0.91; 1.33	0.75	0.22; 2.53	1.01	0.93; 1.10
EGF	0.98	0.89; 1.08	0.96	0.85; 1.09	1.04	0.90; 1.20	0.97	0.81; 1.16
FGF	**1.08**	**1.02; 1.14**	0.93	0.85; 1.03	1.08	0.97; 1.19	1.03	0.93; 1.13
HGF	**1.06**	**1.01; 1.10**	0.96	0.90; 1.03	1.00	0.94; 1.06	1.05	0.93; 1.20
VEGF	**1.09**	**1.02; 1.17**	0.97	0.84; 1.12	1.05	0.89; 1.23	1.05	0.92; 1.19
G-CSF	1.10	0.88; 1.36	1.03	0.74; 1.43	0.96	0.75; 1.24	1.06	0.88; 1.29
GM-CSF	1.02	0.99; 1.06	1.04	0.97; 1.11	1.09	0.98; 1.21	1.00	0.95; 1.05
IL-7	**1.05**	**1.01; 1.08**	0.97	0.88; 1.07	0.99	0.84; 1.16	1.03	0.91; 1.17

Multivariable logistic regression models adjusting for the following variables: site, age at recruitment, gestational age, parity, delivery mode (just for delivery samples) and *P*. *falciparum* infection were estimated. *P*. *vivax* infection cases included those diagnosed by either PCR or microscopy. Odds ratio (OR) per 25% increase in biomarker concentration. Recruitment, N = 275; delivery periphery, N = 199 (infection rates by *Plasmodium spp*. and timepoint in S2 Table). Placenta N = 75 (61 *Pv*-, 14 *Pv*+). Cord N = 82 (57 *Pv*- 25 *Pv*+). In bold if 95% confidence interval (CI) does not include 1. N/A: regression model could not be estimated as all samples considered have the same value for IFN-γ concentration (9.6 pg/mL).

Finally, we investigated the different effect (if any) of submicroscopic and microscopic *Pv* infections, i.e. infection density, on biomarker plasma concentration at recruitment. Results were interpreted based on 95% CI. On the one hand, *Pv* microscopic but not submicroscopic infections were associated with elevated plasma concentrations of proinflammatory biomarkers TNF, IL-1β, IL-6, CXCL8, CCL2, CXCL10, CXCL9; the antiinflammatory IL-10 and IL-1RA; the chemokine CCL5; the T_H_1-related cytokine IL-2R; the T_H_2-related cytokine IL-5; the T_H_17-related cytokine IL-17 and the growth factors VEGF and GM-CSF (Table 4). On the other hand, *Pv* submicroscopic but not microscopic infections were associated with elevated plasma concentrations of biomarkers IL-2, FGF and IL-7 (Table 4). Of note, when we did this stratification, the negative association with CCL11 levels was lost (Table 4).

**Table 4:** Association of plasma biomarker concentration with microscopic and submicroscopic *P*. *vivax* infection.

	RECRUITMENT					DELIVERY				
	Neg	PCR+		Microscopy +		Neg	PCR+		Microscopy +	
	Effect	Effect	95% CI	Effect	95% CI	Effect	Effect	95% CI	Effect	95% CI
TNF	0	0.94	-0.32; 2.20	**1.53**	**0.17; 2.88**	0	0.58	-0.14; 1.31	**1.89**	**0.46; 3.32**
IL-1β	0	1.31	-0.09; 2.71	**2.98**	**1.48; 4.48**	0	0.20	-0.50; 0.90	0.21	-1.18; 1.60
IL-6	0	0.30	-0.77; 1.36	**1.89**	**0.75; 3.03**	0	**-0.68**	**-1.23; -0.13**	0.29	-0.80; 1.38
IL-10	0	0.38	-0.37; 1.14	**1.75**	**0.94; 2.56**	0	-0.22	-0.71; 0.27	**1.17**	**0.19; 2.14**
IL-1RA	0	0.46	-0.19; 1.10	**0.74**	**0.05; 1.43**	0	0.17	-0.34; 0.68	0.07	-0.94; 1.08
TGF-β	0	-0.01	-0.44; 0.42	-0.05	-0.49; 0.39	0	0.04	-0.25; 0.32	0.26	-0.29; 0.81
IFN-α	0	0.26	-0.15; 0.68	0.21	-0.23; 0.65	0	**0.34**	**0.03; 0.65**	0.02	-0.60; 0.64
CXCL8	0	-0.14	-1.16; 0.88	**1.18**	**0.09; 2.28**	0	0.39	-0.67; 1.45	1.13	-0.98; 3.24
CCL3	0	**0.65**	**0.04; 1.26**	**1.15**	**0.50; 1.81**	0	-0.10	-0.48; 0.28	-0.16	-0.92; 0.59
CCL4	0	-0.01	-0.60; 0.57	0.31	-0.32; 0.93	0	-0.26	-0.68; 0.15	-0.66	-1.47; 0.16
CCL2	0	0.62	-0.25; 1.49	**1.76**	**0.82; 2.69**	0	-0.07	-0.63; 0.49	-0.20	-1.31; 0.92
CXCL10	0	0.36	-0.32; 1.04	**1.07**	**0.34; 1.80**	0	-0.1	-0.51; 0.32	0.03	-0.80; 0.85
CXCL9	0	0.19	-0.39; 0.78	**0.67**	**0.04; 1.30**	0	-0.21	-0.75; 0.33	0.36	-0.72; 1.43
CCL11	0	0.03	-0.43; 0.49	-0.43	-0.93; 0.06	0	-0.14	-0.53; 0.24	-0.14	-0.90; 0.63
CCL5	0	0.26	-0.19; 0.70	**0.62**	**0.14; 1.10**	0	0.04	-0.34; 0.42	0.46	-0.30; 1.22
IFN-γ	0	0.00	-0.17; 0.16	0.00	-0.18; 0.18	0	0.02	-0.17; 0.20	-0.10	-0.47; 0.27
IL-12	0	**0.30**	**0.03; 0.57**	**0.38**	**0.09; 0.67**	0	0.17	-0.04; 0.38	0.20	-0.21; 0.62
IL-2	0	**0.75**	**0.08; 1.43**	0.28	-0.45; 1.01	0	-0.14	-0.68; 0.39	-0.80	-1.85; 0.26
IL-15	0	**0.76**	**0.12; 1.40**	**1.01**	**0.32; 1.70**	0	0.14	-0.34; 0.63	-0.36	-1.33; 0.60
IL-2R	0	0.44	-0.10; 0.98	**1.07**	**0.49; 1.65**	0	0.03	-0.30; 0.35	0.13	-0.51; 0.77
IL-4	0	0.02	-0.17; 0.20	-0.05	-0.25; 0.15	0	-0.06	-0.32; 0.21	-0.16	-0.69; 0.37
IL-5	0	0.86	-0.42; 2.13	**1.89**	**0.52; 3.26**	0	0.02	-0.72; 0.76	**1.68**	**0.21; 3.15**
IL-13	0	-0.05	-0.62; 0.53	0.23	-0.38; 0.85	0	-0.05	-0.42; 0.32	-0.32	-1.05; 0.41
IL-17	0	0.55	-0.50; 1.60	**1.28**	**0.16; 2.41**	0	0.57	0.03; 1.12	-0.12	-1.20; 0.96
EGF	0	-0.26	-0.81; 0.28	-0.21	-0.79; 0.38	0	-0.28	-0.63; 0.08	-0.15	-0.85; 0.56
FGF	0	**0.99**	**0.34; 1.64**	0.59	-0.11; 1.28	0	-0.40	-0.93; 0.12	-0.32	-1.36; 0.72
HGF	0	0.90	-0.25; 2.04	0.17	-1.05; 1.39	0	-0.65	-1.38; 0.08	0.13	-1.26; 1.52
VEGF	0	0.07	-0.40; 0.55	**0.98**	**0.48; 1.48**	0	-0.36	-0.73; 0.01	-0.13	-0.87; 0.60
G-CSF	0	0.09	-0.18; 0.35	0.06	-0.22; 0.34	0	-0.09	-0.26; 0.08	0.13	-0.20; 0.47
GM-CSF	0	0.78	-0.46; 2.03	**1.52**	**0.18; 2.86**	0	0.39	-0.40; 1.18	-0.27	-1.81; 1.27
IL-7	0	**1.58**	**0.32; 2.83**	1.18	-0.17; 2.53	0	0.31	-0.52; 1.14	1.55	-0.06; 3.17

Multivariable logistic regression models adjusting for site. CI: confidence interval. Neg: no infection detected by either PCR or microscopy. PCR+: PCR positive and microscopy negative. Microscopy +: smear positive regardless of PCR result. Expected change: change in mean concentration measured in pg/mL. Recruitment N = 76: Neg N = 26; PCR+ N = 36; Microscopy + N = 14. Delivery N = 89: Neg N = 61; PCR+ N = 24; Microscopy+ N = 4. In bold if 95% CI does not include 0.

### Association of *Pv* infection with plasma biomarker concentration at delivery

In PCA analysis at delivery, seven PCs also resulted in eigenvalue>1 (kmo = 0.86, S5 Table). However, regression models showed no association of any PC with *Pv* infection at delivery (S6 Table). We did not observe differences between *Pv*-infected and uninfected women in plasma biomarker levels in the crude analysis (not shown) at any compartment. In the adjusted analysis, we observed a positive association of *Pv* infection with IFN-α and IL-12 peripheral plasma concentration and with IL-10 placental plasma concentration (Table 3). After stratifying by *Plasmodium* infection density, submicroscopic infection was associated with increased peripheral concentrations of IFN-α and decreased concentrations of IL-6, while microscopic infections were associated with elevated levels of TNF, IL-10 and IL-5. Moreover, microscopic infections were associated with increased concentrations of TNF and IL-5 (Table 4).

### Plasma biomarker concentration and delivery outcomes

Hb levels at delivery were positively associated with CCL11 and FGF peripheral plasma concentrations at recruitment (Table 5) and with CXCL9 placental plasma concentration (Table 6), and negatively associated with IL-1RA and G-CSF cord plasma concentrations (Table 6). Birth weight showed no association with any biomarker at recruitment (Table 5), and it was negatively associated with peripheral IL-4 concentration at delivery (Table 6).

**Table 5:** Association of biomarkers at recruitment with hemoglobin levels at delivery and birth weight.

	Hemoglobin (g/dL)		Birth weight (g)	
	Effect	95% CI	Effect	95% CI
TNF	0.01	-0.02; 0.05	-3.83	-11.40; 3.75
IL-1β	0.01	-0.03; 0.04	0.38	-7.23; 8.00
IL-6	-0.02	-0.07; 0.03	-8.75	-21.92; 4.41
IL-10	0.00	-0.06; 0.07	-16.00	-32.73; 0.73
IL-1RA	0.01	-0.06; 0.08	-3.38	-21.29; 14.53
TGF-β	-0.08	-0.20; 0.04	-1.15	-32.95; 30.65
IFN-α	-0.01	-0.12; 0.11	-24.09	-52.01; 3.82
CXCL8	0.01	-0.03; 0.06	-6.34	-16.67; 3.98
CCL3	0.00	-0.08; 0.09	-3.60	-24.23; 17.03
CCL4	0.02	-0.04; 0.08	-3.48	-18.15; 11.19
CCL2	0.03	-0.06; 0.13	1.47	-22.06; 25.00
CXCL10	-0.03	-0.12; 0.06	-15.18	-37.20; 6.85
CXCL9	-0.01	-0.09; 0.08	-6.56	-27.38; 14.25
CCL11	**0.15**	**0.03; 0.26**	-9.84	-38.48; 18.80
CCL5	0.00	-0.12; 0.11	1.41	-26.42; 29.23
IFN-γ	-0.15	-0.49; 0.19	-50.53	-135.09; 34.03
IL-12	0.04	-0.17; 0.24	4.84	-44.48; 54.16
IL-2	0.05	-0.01; 0.11	12.87	-2.32; 28.06
IL-15	0.01	-0.06; 0.07	3.55	-12.91; 20.00
IL-2R	-0.02	-0.11; 0.07	-7.58	-30.77; 15.60
IL-4	0.00	-0.18; 0.17	-18.35	-62.30; 25.59
IL-5	0.02	-0.02; 0.06	-4.17	-12.20; 3.87
IL-13	0.01	-0.08; 0.11	-3.97	-26.94; 19.00
IL-17	0.02	-0.02; 0.06	1.56	-7.41; 10.53
EGF	0.05	-0.02; 0.12	10.98	-6.24; 28.20
FGF	**0.06**	**0.00; 0.12**	3.26	-11.52; 18.04
HGF	0.01	-0.03; 0.05	-2.61	-12.52; 7.30
VEGF	0.00	-0.10; 0.11	-17.65	-35.44; 0.13
G-CSF	0.02	-0.02; 0.06	-6.24	-14.68; 2.20
GM-CSF	-0.01	-0.19; 0.16	-13.46	-54.75; 27.84
IL-7	0.03	-0.01; 0.07	0.10	-8.52; 8.72

Multivariable linear regression models adjusting for the following variables: site, age at recruitment, hemoglobin (Hb) at recruitment for analysis of Hb at delivery, gravidity, gestational age, delivery mode, and *P*. *falciparum* and *P*. *vivax* infection. Effect: change in Hb levels (g/dL) or birth weight (g) per 25% increase in biomarker concentration. N = 145. In bold if 95% confidence interval (CI) does not include 0.

**Table 6:** Association of biomarkers at delivery with hemoglobin levels at delivery and birth weight.

.	Periphery				Placenta				Cord			
	Hemoglobin		Birth weight		Hemoglobin		Birth weight		Hemoglobin		Birth weight	
	Effect	95%CI	Effect	95%CI	Effect	95%CI	Effect	95%CI	Effect	95%CI	Effect	95%CI
TNF	0.03	-0.04; 0.10	0.19	-18.88; 19.27	0.01	-0.15; 0.17	26.49	-6.86; 59.85	0.00	-0.05; 0.05	-4.84	-16.71; 7.03
IL-1β	-0.03	-0.09; 0.03	-6.18	-23.11; 10.75	0.00	-0.15; 0.14	22.00	-1.36; 45.37	0.00	-0.04; 0.05	-0.26	-10.49; 9.97
IL-6	0.01	-0.04; 0.06	5.35	-7.69; 18.38	0.03	-0.03; 0.09	10.48	-1.89; 22.86	-0.03	-0.08; 0.02	-9.03	-20.49; 2.44
IL-10	-0.03	-0.10; 0.04	-14.67	-33.31; 3.97	0.11	-0.14; 0.36	26.79	-27.44; 81.03	-0.03	-0.71; 0.64	-9.31	-169.30; 150.69
IL-1RA	-0.03	-0.09; 0.03	-7.08	-23.32; 9.17	0.10	-0.01; 0.21	10.36	-13.19; 33.90	**-0.15**	**-0.29; -0.01**	-25.49	-58.66; 7.68
TGF-β	-0.02	-0.18; 0.13	-16.36	-58.59; 25.88	0.00	-0.10; 0.10	3.58	-17.84; 25.00	-0.06	-0.19; 0.07	11.11	-20.40; 42.62
IFN-α	-0.02	-0.13; 0.08	-22.77	-50.47; 4.93	0.15	-0.05; 0.35	29.95	-11.01; 70.92	-0.05	-0.40; 0.31	4.66	-77.58; 86.89
CXCL8	0.00	-0.03; 0.03	0.69	-7.62; 8.99	0.03	-0.03; 0.08	8.70	-3.21; 20.60	-0.03	-0.08; 0.02	-9.25	-20.08; 1.57
CCL3	0.07	-0.01; 0.14	-6.41	-26.16; 13.34	0.07	-0.07; 0.22	22.50	-7.74; 52.74	-0.15	-0.32; 0.03	-4.51	-45.79; 36.77
CCL4	0.01	-0.06; 0.08	-0.73	-19.43; 17.97	0.00	-0.10; 0.10	10.83	-9.85; 31.50	-0.01	-0.08; 0.06	-13.29	-29.61; 3.03
CCL2	0.01	-0.04; 0.06	-1.50	-15.10; 12.09	0.04	-0.06; 0.14	6.60	-15.10; 28.30	-0.02	-0.09; 0.05	-11.58	-27.52; 4.35
CXCL10	0.05	-0.03; 0.13	14.89	-7.40; 37.18	**0.08**	**0.00; 0.17**	3.02	-15.73; 21.78	0.02	-0.11; 0.15	-17.21	-47.33; 12.91
CXCL9	0.02	-0.05; 0.08	2.32	-14.58; 19.21	**0.09**	**0.01; 0.17**	-0.86	-19.02; 17.30	-0.02	-0.11; 0.07	-7.02	-27.43; 13.39
CCL11	0.01	-0.08; 0.10	3.75	-20.49; 27.99	0.02	-0.13; 0.17	15.21	-17.60; 48.03	0.10	-0.06; 0.27	33.55	-4.87; 71.98
CCL5	-0.05	-0.15; 0.05	19.81	-5.78; 45.41	0.03	-0.06; 0.12	6.22	-14.02; 26.46	-0.04	-0.22; 0.14	16.95	-23.90; 57.81
IFN-γ	0.05	-0.15; 0.24	-8.66	-60.06; 42.75	-11.83	-62.25; 38.60	-1625.89	-1.3e+04; 9369.71	4.10	-0.39; 8.59	258.64	-857.77; 1375.05
IL-12	-0.02	-0.17; 0.13	-5.63	-44.85; 33.59	0.01	-0.17; 0.18	36.44	-1.27; 74.16	-0.01	-0.30; 0.29	-9.16	-78.97; 60.64
IL-2	**-0.07**	**-0.13; -0.01**	1.96	-13.98; 17.89	0.09	-0.05; 0.23	-7.16	-38.20; 23.88	0.00	-0.08; 0.08	12.17	-5.97; 30.31
IL-15	-0.06	-0.14; 0.02	6.61	-13.80; 27.02	0.09	-0.02; 0.20	6.93	-17.89; 31.75	0.02	-0.12; 0.15	-21.09	-52.08; 9.90
IL-2R	-0.03	-0.13; 0.06	-10.66	-35.41; 14.09	0.15	-0.04; 0.34	13.51	-28.76; 55.79	-0.04	-0.23; 0.15	-11.77	-57.33; 33.79
IL-4	-0.02	-0.15; 0.11	**-43.01**	**-76.74; -9.27**	N/A regression model		N/A regression model		2.56	-0.20; 5.33	2.56	-0.25; 5.36
IL-5	0.01	-0.05; 0.06	-9.12	-23.96; 5.72	-0.10	-0.31; 0.11	5.65	-40.90; 52.19	-0.02	-0.07; 0.03	5.30	-6.61; 17.21
IL-13	0.03	-0.04; 0.11	-17.75	-37.69; 2.19	0.01	-0.21; 0.24	-2.60	-51.12; 45.93	0.01	-0.33; 0.36	3.93	-76.19; 84.05
IL-17	-0.01	-0.12; 0.10	-22.26	-51.12; 6.59	0.18	-0.27; 0.63	60.09	-33.19; 153.37	0.04	-0.06; 0.14	-8.10	-31.69; 15.49
EGF	-0.07	-0.15; 0.02	-5.31	-27.93; 17.32	0.08	-0.04; 0.21	1.85	-26.51; 30.20	-0.01	-0.18; 0.15	-18.33	-56.15; 19.48
FGF	-0.04	-0.10; 0.02	5.93	-9.57; 21.43	0.05	-0.05; 0.15	-5.45	-27.93; 17.04	0.03	-0.08; 0.14	-6.93	-33.37; 19.51
HGF	0.00	-0.05; 0.04	-7.04	-19.74; 5.66	0.00	-0.06; 0.07	-1.62	-15.52; 12.27	-0.08	-0.20; 0.04	-20.29	-48.62; 8.04
VEGF	-0.01	-0.09; 0.08	6.27	-16.05; 28.60	0.11	-0.01; 0.23	16.61	-9.45; 42.67	-0.08	-0.20; 0.03	-26.18	-52.87; 0.50
G-CSF	-0.06	-0.21; 0.09	-21.01	-60.86; 18.84	0.06	-0.08; 0.20	20.24	-9.51; 49.98	**-0.21**	**-0.37; -0.04**	20.83	-19.02; 60.69
GM-CSF	-0.01	-0.05; 0.04	-5.97	-18.69; 6.75	-0.03	-0.26; 0.20	31.96	-18.02; 81.94	0.01	-0.04; 0.06	3.61	-7.06; 14.28
IL-7	0.00	-0.05; 0.06	-3.19	-17.42; 11.04	-0.01	-0.15; 0.12	13.18	-15.61; 41.97	-0.04	-0.16; 0.08	-15.21	-42.00; 11.58

Multivariable linear regression models adjusting for the following variables: site, age at recruitment, hemoglobin (Hb) at recruitment for analysis of Hb at delivery, Hb at delivery for analysis of birth weight (BW), gravidity, delivery mode, *P*. *falciparum* and *P*. *vivax* infection. Effect: change in Hb levels (mg/dL) or BW (g) per 25% increase in biomarker concentration. Periphery N = 188; Placenta N = 75; Cord N = 81; In bold if 95% confidence interval (CI) does not include 0. All samples considered have the same value for IL-4 concentration in plasma (pg/ml). N/A regression model: model could not be estimated as all samples considered have the same value for IL-4 concentration (38.96 pg/mL).

## Discussion

We report an exhaustive profiling of plasma biomarkers including cytokines, chemokines and growth factors in malaria in pregnancy caused by *Pv*, and their association with poor delivery outcomes. We separated the analysis of samples obtained at the first antenatal visit from the ones at delivery, as previous analyses in this cohort showed differences in most biomarker concentrations between recruitment and delivery [8]. However, recruitment samples, which were collected at first, second and third trimester of pregnancy, were not further categorized because the correlation between women’s gestational age and biomarker concentration in plasma was low in all cases in this cohort [8].

It is well known that *Plasmodium spp*. infection is accompanied by an inflammatory response that seems to correlate with severity of malaria disease [9,13–18]. Also, placental inflammation has been shown in *Pf* malaria in pregnancy and linked to poor delivery outcomes [19–22]. However, the peripheral compartment in malaria during pregnancy has been less well studied, especially for *Pv* malaria in pregnancy. Here we showed that, in pregnant women at enrolment, *Pv* infection is associated with a broad proinflammatory response. First, in the exploratory PCA analysis we showed that two of the three clusters of cytokines associated with *Pv* infection were mainly proinflammatory: the PC3, composed by CXCL8, CCL4 and CCL3; and the PC5, composed by CXCL10, IL-6, CCL2 and IL-10. In agreement with this, the crude and adjusted analyses showed positive associations of IL-6, IL-1β, CXCL8, CCL3, CCL4, CCL2 and CXCL10 with *Pv* infection. *Pv* microscopic but not submicroscopic infections accounted for this inflammatory response. Another study in India has recently shown that women with *Pv* malaria in pregnancy have more IL-6, TNF and IL-1β in peripheral plasma than uninfected pregnant women [10].

However, all the above associations of infection with inflammation were lost at delivery. No PCs and no individual proinflammatory biomarker showed any association with *Pv* infection at delivery. Only microscopic infections at delivery showed a positive association with TNF levels. We [8] and others [23,24] have shown that labor is accompanied by a peripheral proinflammatory response which may have masked any differences in inflammatory biomarker concentrations between infected and uninfected women. Moreover, there is controversy about *Pv* cytoadhesion to placenta but we have reported placental *Pv* monoinfections with no signs of placental inflammation [25].

Despite the strong evidence of a potent inflammatory response triggered by *Pv* infection during pregnancy, our results did not show an impact of inflammation at recruitment on delivery outcomes. Moreover, in this cohort no poor delivery outcomes were attributed to *Pv* infection during pregnancy, except for anemia in symptomatic *Pv*-infected women [3]. According to our data, we propose that an antiinflammatory response could be compensating the excessive inflammation. Thus, IL-10 (antiinflammatory cytokine) clustered with CXCL10, IL-6 and CCL2 (all proinflammatory biomarkers) in the PC5, which was associated with *Pv* infection at recruitment. Moreover, we showed a positive association of *Pv* infection with peripheral IL-10 concentration at recruitment, peripheral IL-10 at delivery (association only with microscopic infections) and placental IL-10 concentration at delivery. In addition, cord IL-1RA levels showed a negative association with Hb levels at delivery. Others have shown that *Pv* infection induces a proinflammatory response associated with an immunomodulatory profile mediated by IL-10 and TGF-β [17] and production of IL-10 and expansion of regulatory T cells [26] in non-pregnant individuals.

We also studied T_H_-related biomarkers and found that while *Pv* infection was vaguely associated with T_H_2-related IL-5, infection was consistently and positively associated with T_H_1 cytokines (but not IFN-γ) in the PCA analysis (where IL-12 clustered with IL-2R in the PC7), as well as the crude and the adjusted analyses. Among them, the strongest association was observed with IL-12 plasma concentration, the key cytokine in T_H_1 differentiation. Moreover, CCL11, which has been associated to T_H_2-responses in allergic reactions and is able to recruit T_H_2 lymphocytes [27], showed a negative association with *Pv* infection, supporting the hypothesis that *Pv* malaria in pregnancy triggers a T_H_1 response. Elevated levels of IFN-γ have been positively, negatively and not associated with placental *Pf* malaria (reviewed in [28]) and the role of the T_H_1 arm in malaria-related poor delivery outcomes is also controversial [21,29].

According to our present and previous data, we propose that failing to mount a T_H_1 response might worsen delivery outcomes, as we showed here that T_H_2-type IL-4 peripheral plasma concentration at delivery was negatively associated with birth weight, while previous results of a flow cytometry analysis of PNG women in this cohort showed a protective role of circulating T_H_1 cells (CD3^+^-CD4^+^-IFN-γ^+^-IL-10^-^) with birth weight [6].

CCL11, a chemokine poorly studied in the context of malaria, was the only biomarker among the 31 studied to show a negative association (OR<1) with *Pv* infection, although the associations were lost when stratifying by infection density. We had previously shown that non-pregnant women heavily exposed to malaria had lower levels of circulating CCL11 than malaria-naïve controls [7]. Thus it seems that (low) CCL11 concentration could be used as a marker of malaria infection/exposure. Our analysis supports this finding, as higher CCL11 plasma concentration at recruitment was associated with higher Hb levels at delivery, and we had established previously that clinical *Pv* infection is associated with maternal anemia [3]. However, from this study we cannot determine whether this association is causative and/or what role (if any) CCL11 has on *Pv* infection.

Our study presents some limitations. Blood samples were collected in heparin vacutainers, therefore we cannot rule out contamination of plasma by platelet-derived factors. Also, the restricted number of samples with quantified parasitemia, the low concentration of some cytokines, and the number of women who were lost to follow up, prevented us from performing more detailed analyses on the relationship between certain cytokines and malaria prospectively. Moreover, we did not collect information regarding important and prevalent infectious diseases in some of the study sites, such as helminth infections. However, this may not be considered as a bias in the CCL11 analysis, as helminths would actually provoke an increase in CCL11 while we observe a decrease in this chemokine associated to malaria.

In conclusion, data show that while T_H_1 and proinflammatory responses are dominant during *Pv* infection in pregnancy, antiinflammatory cytokines may compensate excessive inflammation avoiding poor delivery outcomes, and skewness towards a T_H_2 response may trigger worse delivery outcomes. CCL11, a chemokine largely neglected in the field of malaria, emerges as an important marker of exposure or mediator in this condition.

## Supporting information

S1 Fig Flow chart of sample selection for the study. (DOCX)

S1 Table Upper and lower values of the biomarker standard curves. (DOCX)

S2 Table *Plasmodium* infection case number by country. 1: n (percentage).
(DOCX)

S3 Table Principal component analysis of biomarkers at recruitment. PC: principal component. N = 301.
(DOCX)

S4 Table Association of principal components with *P*. *vivax* infection at recruitment. After varimax rotation, principal component scores were predicted and used as independent variables in logistic regression models. OR: odd ratio. CI: confidence interval. In bold if p<0.05.
(DOCX)

S5 Table Principal component analysis of biomarkers at delivery. PC: principal component. N = 281.
(DOCX)

S6 Table Association of principal components with *P*. *vivax* infection at delivery. After varimax rotation, principal component scores were predicted and used as independent variables in logistic regression models. OR: odd ratio. CI: confidence interval.
(DOCX)
