# Supplementary material for: Cytokine signatures of Plasmodium vivax infection during pregnancy and delivery outcomes
Source: PLoS Negl Trop Dis. 2020 May 4;14(5):e0008155. doi: 10.1371/journal.pntd.0008155 (PMC7224570; doi:10.1371/journal.pntd.0008155)
Supplement: S1 Fig — (DOCX) [file pntd.0008155.s001.docx]

**S1 Fig. Flow chart of sample selection for the study.**

235 random samples collected at recruitment

129 peripheral plasmas collected at delivery and paired to recruitment samples

106 peripheral plasmas collected at delivery and not paired to recruitment samples

49 *Plasmodium vivax* positive samples collected at recruitment

62 *P. vivax* negative samples collected at recruitment

18 *P. vivax* positive samples collected at delivery

7 *P. vivax* negative samples collected at delivery

470 samples

341 women

606 samples

447 women

144 samples from placental blood

125 peripheral plasmas collected at delivery and paired to the placental samples

112 cord plasmas paired to peripheral plasmas collected at delivery

987 samples

572 women
