# Supplementary material for: Cytokine signatures of Plasmodium vivax infection during pregnancy and delivery outcomes
Source: PLoS Negl Trop Dis. 2020 May 4;14(5):e0008155. doi: 10.1371/journal.pntd.0008155 (PMC7224570; doi:10.1371/journal.pntd.0008155)
Supplement: S1 Table — (DOCX) [file pntd.0008155.s002.docx]

**S1 Table. Upper and lower values of the biomarker standard curves.**

| Biomarker | Std 1 (upper) pg/mL | Std 7 (lower) pg/mL |
| --- | --- | --- |
| TNF | 8700 | 12 |
| IL1B | 8300 | 11 |
| IL-6 | 4150 | 6 |
| IL-10 | 21900 | 30 |
| TGF-β | 2500 | 39 |
| IL-1RA | 28000 | 38 |
| IFN-α | 12900 | 18 |
| CXCL8 | 12800 | 18 |
| CCL3 | 25000 | 34 |
| CCL4 | 7200 | 10 |
| CCL2 | 6100 | 8 |
| CXCL10 | 900 | 1 |
| CCL11 | 2900 | 4 |
| CCL5 | 3500 | 5 |
| CXCL9 | 2400 | 3 |
| IFN-γ | 10500 | 14 |
| IL-12 | 6500 | 9 |
| IL-2 | 1200 | 1.6 |
| IL-15 | 24300 | 33 |
| IL-2R | 21000 | 29 |
| IL-4 | 42600 | 58 |
| IL-5 | 2330 | 3 |
| IL-13 | 20300 | 28 |
| IL-17 | 38800 | 53 |
| EGF | 12000 | 16 |
| FGF | 2400 | 3 |
| HGF | 7900 | 11 |
| VEGF | 4400 | 6 |
| G-CSF | 70000 | 96 |
| GM-CSF | 17350 | 24 |
| IL-7 | 9800 | 13 |
