# Supplementary material for: Cytokine signatures of Plasmodium vivax infection during pregnancy and delivery outcomes
Source: PLoS Negl Trop Dis. 2020 May 4;14(5):e0008155. doi: 10.1371/journal.pntd.0008155 (PMC7224570; doi:10.1371/journal.pntd.0008155)
Supplement: S2 Table — 1: n (percentage). (DOCX) [file pntd.0008155.s003.docx]

**S2 Table. *Plasmodium* infection case number by country**

| **RECRUITMENT** |  | **Brazil** | **Colombia** | **Guatemala** | **India** | **PNG** |
| --- | --- | --- | --- | --- | --- | --- |
| *P. vivax* (by microscopy)^1^ | negative | 73 (91%) | 103 (90%) | 101 (99%) | 57 (100%) | 233 (94%) |
|  | positive | 4 (5%) | 9 (8%) | 1 (1%) | 0 (0%) | 10 (4%) |
| *P. falciparum* (by microscopy) | negative | 80 (100%) | 108 (94%) | 102 (100%) | 57 (100%) | 230 (93%) |
|  | positive | 0 (0%) | 6 (5%) | 0 (0%) | 0 (0%) | 13 (5%) |
| *P. vivax* (by PCR) | negative | 17 (77%) | 12 (80%) | 8 (62%) | 4 (100%) | 27 (42%) |
|  | positive | 5 (23%) | 3 (20%) | 5 (38%) | 0 (0%) | 37 (58%) |
| *P. falciparum* (by PCR) | negative | 22 (100%) | 14 (93%) | 12 (92%) | - | 52 (81%) |
|  | positive | 0 (0%) | 1 (7%) | 1 (8%) | - | 12 (19%) |
| **DELIVERY** |  | **Brazil** | **Colombia** | **Guatemala** | **India** | **PNG** |
| *P. vivax* (by microscopy) | negative | 43 (100%) | 76 (95%) | 73 (99%) | 41 (100%) | 177 (97%) |
|  | positive | 0 (0%) | 2 (3%) | 1 (1%) | 0 (0%) | 5 (3%) |
| *P. falciparum* (by microscopy) | negative | 43 (100%) | 78 (98%) | 74 (100%) | 41 (100%) | 178 (98%) |
|  | positive | 0 (0%) | 1 (1%) | 0 (0%) | 0 (0%) | 4 (2%) |
| *P. vivax* (by PCR) | negative | 26 (100%) | 39 (85%) | 7 (44%) | 6 (100%) | 64 (73%) |
|  | positive | 0 (0%) | 7 (15%) | 9 (56%) | 0 (0%) | 24 (27%) |
| P*. falciparum* (by PCR) | negative | 26 (100%) | 42 (91%) | 16 (100%) | - | 84 (95%) |
|  | positive | 0 (0%) | 4 (9%) | 0 (0%) | - | 4 (5%) |

1: n (percentage). Two women at recruitment and one woman at delivery were coinfected with *P. vivax* and P. *falciparum*, as diagnosed by PCR.
