# Supplementary material for: Cytokine signatures of Plasmodium vivax infection during pregnancy and delivery outcomes
Source: PLoS Negl Trop Dis. 2020 May 4;14(5):e0008155. doi: 10.1371/journal.pntd.0008155 (PMC7224570; doi:10.1371/journal.pntd.0008155)
Supplement: S3 Table — PC: principal component. N = 301. (DOCX) [file pntd.0008155.s004.docx]

**S3 Table. Principal component analysis of biomarkers at recruitment**

| Component | Eigenvalue | Proportion | Cumulative |
| --- | --- | --- | --- |
| PC1 | 8.71961 | 0.2907 | 0.2907 |
| PC2 | 3.73127 | 0.1244 | 0.415 |
| PC3 | 2.30653 | 0.0769 | 0.4919 |
| PC4 | 1.74482 | 0.0582 | 0.5501 |
| PC5 | 1.43159 | 0.0477 | 0.5978 |
| PC6 | 1.25066 | 0.0417 | 0.6395 |
| PC7 | 1.13736 | 0.0379 | 0.6774 |
| PC8 | 0.953419 | 0.0318 | 0.7092 |
| PC9 | 0.860163 | 0.0287 | 0.7378 |
| PC10 | 0.795526 | 0.0265 | 0.7644 |
| PC11 | 0.724458 | 0.0241 | 0.7885 |
| PC12 | 0.65184 | 0.0217 | 0.8102 |
| PC13 | 0.643592 | 0.0215 | 0.8317 |
| PC14 | 0.552697 | 0.0184 | 0.8501 |
| PC15 | 0.472818 | 0.0158 | 0.8659 |
| PC16 | 0.436078 | 0.0145 | 0.8804 |
| PC17 | 0.39223 | 0.0131 | 0.8935 |
| PC18 | 0.382647 | 0.0128 | 0.9062 |
| PC19 | 0.370942 | 0.0124 | 0.9186 |
| PC20 | 0.344186 | 0.0115 | 0.9301 |
| PC21 | 0.311788 | 0.0104 | 0.9405 |
| PC22 | 0.295509 | 0.0099 | 0.9503 |
| PC23 | 0.260048 | 0.0087 | 0.959 |
| PC24 | 0.236984 | 0.0079 | 0.9669 |
| PC25 | 0.217668 | 0.0073 | 0.9741 |
| PC26 | 0.201747 | 0.0067 | 0.9809 |
| PC27 | 0.181193 | 0.006 | 0.9869 |
| PC28 | 0.161513 | 0.0054 | 0.9923 |
| PC29 | 0.11757 | 0.0039 | 0.9962 |
| PC30 | 0.113536 | 0.0038 | 1 |

PC: principal component. N=301
