# Supplementary material for: Cytokine signatures of Plasmodium vivax infection during pregnancy and delivery outcomes
Source: PLoS Negl Trop Dis. 2020 May 4;14(5):e0008155. doi: 10.1371/journal.pntd.0008155 (PMC7224570; doi:10.1371/journal.pntd.0008155)
Supplement: S4 Table — After varimax rotation, principal component scores were predicted and used as independent variables in logistic regression models. OR: odd ratio. CI: confidence interval. In bold if p<0.05. (DOCX) [file pntd.0008155.s005.docx]

**S4 Table. Association of principal components with *P. vivax* infection at recruitment.**

|  | 0R | 95% CI | p-value |
| --- | --- | --- | --- |
| PC1 | 1.13 | 0.99: 1.30 | 0.073 |
| PC2 | 1.10 | 0.96: 1.25 | 0.171 |
| **PC3** | **1.50** | **1.28: 1.75** | **<0.001** |
| PC4 | 1.07 | 0.92: 1.26 | 0.369 |
| **PC5** | **1.50** | **1.26: 1.79** | **<0.001** |
| PC6 | 1.06 | 0.85: 1.32 | 0.614 |
| **PC7** | **1.64** | **1.29: 2.09** | **<0.001** |

After varimax rotation, principal component scores were predicted and used as independent variables in logistic regression models. OR: odd ratio. CI: confidence interval. In bold if p<0.05.
