# Supplementary material for: Cytokine signatures of Plasmodium vivax infection during pregnancy and delivery outcomes
Source: PLoS Negl Trop Dis. 2020 May 4;14(5):e0008155. doi: 10.1371/journal.pntd.0008155 (PMC7224570; doi:10.1371/journal.pntd.0008155)
Supplement: S5 Table — PC: principal component. N = 281. (DOCX) [file pntd.0008155.s006.docx]

**S5 Table. Principal component analysis of biomarkers at delivery**

| Component | Eigenvalue | Proportion | Cumulative |
| --- | --- | --- | --- |
| PC1 | 10.1755 | 0.3392 | 0.3392 |
| PC2 | 2.79708 | 0.0932 | 0.4324 |
| PC3 | 1.98684 | 0.0662 | 0.4986 |
| PC4 | 1.77394 | 0.0591 | 0.5578 |
| PC5 | 1.53229 | 0.0511 | 0.6089 |
| PC6 | 1.20693 | 0.0402 | 0.6491 |
| PC7 | 1.10766 | 0.0369 | 0.686 |
| PC8 | 0.961594 | 0.0321 | 0.7181 |
| PC9 | 0.864747 | 0.0288 | 0.7469 |
| PC10 | 0.818033 | 0.0273 | 0.7742 |
| PC11 | 0.728892 | 0.0243 | 0.7984 |
| PC12 | 0.590741 | 0.0197 | 0.8181 |
| PC13 | 0.557639 | 0.0186 | 0.8367 |
| PC14 | 0.513079 | 0.0171 | 0.8538 |
| PC15 | 0.499919 | 0.0167 | 0.8705 |
| PC16 | 0.485646 | 0.0162 | 0.8867 |
| PC17 | 0.455137 | 0.0152 | 0.9019 |
| PC18 | 0.438397 | 0.0146 | 0.9165 |
| PC19 | 0.367023 | 0.0122 | 0.9287 |
| PC20 | 0.34874 | 0.0116 | 0.9403 |
| PC21 | 0.292522 | 0.0098 | 0.9501 |
| PC22 | 0.236116 | 0.0079 | 0.9579 |
| PC23 | 0.228749 | 0.0076 | 0.9656 |
| PC24 | 0.199158 | 0.0066 | 0.9722 |
| PC25 | 0.174719 | 0.0058 | 0.978 |
| PC26 | 0.160706 | 0.0054 | 0.9834 |
| PC27 | 0.150978 | 0.005 | 0.9884 |
| PC28 | 0.134262 | 0.0045 | 0.9929 |
| PC29 | 0.117397 | 0.0039 | 0.9968 |
| PC30 | 0.0956001 | 0.0032 | 1 |

**.**

PC: principal component. N=281
