# Supplementary material for: Cytokine signatures of Plasmodium vivax infection during pregnancy and delivery outcomes
Source: PLoS Negl Trop Dis. 2020 May 4;14(5):e0008155. doi: 10.1371/journal.pntd.0008155 (PMC7224570; doi:10.1371/journal.pntd.0008155)
Supplement: S6 Table — After varimax rotation, principal component scores were predicted and used as independent variables in logistic regression models. OR: odd ratio. CI: confidence interval. (DOCX) [file pntd.0008155.s007.docx]

**S6 Table. Association of principal components with *P. vivax* infection at delivery.**

|  | 0R | 95% CI | p-value |
| --- | --- | --- | --- |
| PC1 | 1.00 | 0.87: 1.15 | 0.983 |
| PC2 | 0.97 | 0.81: 1.17 | 0.755 |
| PC3 | 1.15 | 0.98: 1.35 | 0.080 |
| PC4 | 1.03 | 0.84: 1.26 | 0.783 |
| PC5 | 1.11 | 0.91: 1.35 | 0.298 |
| PC6 | 0.85 | 0.62: 1.15 | 0.290 |
| PC7 | 1.25 | 0.93: 1.67 | 0.138 |

After varimax rotation, principal component scores were predicted and used as independent variables in logistic regression models. OR: odd ratio. CI: confidence interval.
